# The advancement of primary care dentistry in Hungary: progress of the dental cluster model since 2021 legislation

**DOI:** 10.3389/fpubh.2025.1528433

**Published:** 2025-01-22

**Authors:** András Sztrilich, Csilla Kaposvári, Gergő Túri, Rita Teller, István Vingender

**Affiliations:** ^1^Doctoral School of Health Sciences, Faculty of Health Sciences, Semmelweis University, Budapest, Hungary; ^2^Department of Preventive Medicine and Public Health, Semmelweis University, Budapest, Hungary; ^3^Synthesis Health Research Foundation, Budapest, Hungary; ^4^Faculty of Health Sciences, Doctoral School, University of Pécs, Pécs, Hungary; ^5^Epidemiology and Surveillance Centre, Semmelweis University, Budapest, Hungary; ^6^Doctoral School of Health Sciences, University of Debrecen, Debrecen, Hungary; ^7^Faculty of Humanities, Eötvös Loránd University, Budapest, Hungary

**Keywords:** dental services, cluster practice, group practice, primary care, Hungary, finance, regulation, network

## Abstract

**Background:**

Considerable changes in health policy in the area of publicly funded primary dental care in Hungary over the past 25 years have shaped primary care dentists’ financing, training and working conditions and the forms of association between dental providers. This study aims to describe the advancement of primary care dentistry in Hungary and the progress of the dental cluster model since the 2021 legislation.

**Methods:**

A mixed method study was designed that included a scoping review of the literature in December 2023 to collect information on the health policy developments of Hungary’s publicly funded primary dental care system; (b) secondary data analysis was carried out to assess the development of primary care dentistry and the formation and operation of dental cluster practices in Hungary.

**Results:**

Primary care dental clusters in Hungary were established to allow active professional cooperation within the dental clusters to improve the population’s access to preventive dental services and the quality of services. From its legislative base until December 31, 2023, 74 primary dental clusters were formed nationwide, representing 30% of dental practices. On average, the primary dental clusters contain nine practices. There is an uneven geographical distribution of primary dental clusters by counties. Up to the date of this research, primary care dental clusters were set up exclusively in the form of consortia. Adult and mixed dental practices, practices from county towns, county capitals and the capital city, as well as practices from districts with more dentists in the proportion of the population were more likely to join a dental group practice.

**Conclusion:**

Monitoring and evaluating of the operation, performance and impact of primary care dental clusters in the future will provide important information about the Hungarian model.

## 1 Introduction

### 1.1 Background

Over the past 25 years, there have been considerable changes in health policy in Hungary’s publicly funded primary dental care. These legislative and methodological shifts have affected primary care dentists’ financing, training, working conditions and the forms of association between dental providers.

### 1.2 Background information on the Hungarian health system

Healthcare expenditure in Hungary is less than the EU average (in 2021, EU: 11.0%, Hungary 7.4% of its GDP) (1). The Hungarian health system has a single health insurance fund that provides almost universal population coverage (95%) (2). Dental services are included under the statutory benefits package but not fully covered (1, 3). Publicly financed dental services are provided in service districts with territorial coverage and have primary and specialized segments (4). Despite the availability of publicly covered services, out-of-pocket expenses for health care in Hungary are relatively high: 25% of the total health care expenditure (that is higher than the EU average of 15%) of which dental care accounted for 12% in Hungary (1, 5). In 2022, 2.2% of Hungarians aged 16 years or over reported an unmet need for a dental examination or treatment (EU average: 4.8%) (6).

According to the latest European Health Interview Survey, half of the Hungarian population (aged 15 years or over) rated their dental and oral health as “good or very good,” while one-fifth as “bad or very bad” in 2019 (7). Dental diseases are prevalent among the Hungarian population. Two-thirds of the population had dental fillings, 29% had teeth with cavities, 17% had bleeding gums when brushing, and 8% had loose or mobile teeth. 45% of people aged 15 years or older and 77% of those older than 60 had dentures (7). The estimated prevalence of severe periodontal disease (among people aged 15 years or older) in Hungary was 8.6%, one of the lowest in the WHO European Region. On the other hand, the estimated incidence of lip and oral cavity cancers in people of all ages was the highest in Hungary (age-standardized rate: 6.3/100000 population) among the countries of the WHO European Region in 2020 (8). As for healthcare utilization, less than half of the population (48% of women, 44.2% of men) visited a dentist in the year preceding the survey in 2019 (9).

### 1.3 Access to dental care in Hungary

Since the early 1990s in Hungary, municipalities have been responsible for providing primary care services (such as GP care, primary dental care, school health care and public health services) to the population of their settlements (10, 11). The National Directorate General of Hospitals (NDGH) established primary dental care districts with the involvement of the municipalities based on Act CXXIII of 2015 on primary health care (12). Public primary dental care can be provided in two types of practices (adult, and mixed–adult and pediatric) (13). Primary dental care for under 18 years of age is provided by mixed dental practices or school health services. Municipalities may contract to provide services directly, for example, through a health care provider owned/operated by the municipality or indirectly through a self-employed dentist or a non-municipal-owned economic entity according to Act C of 2020 (14). In these contracts, municipalities and dentists specify, among other things, the area and population of the district to be covered, the range of services to be provided, and the office hours (11, 15). Municipalities would also be responsible for providing the infrastructure and equipment needed for dental practice operations. However, in reality, practices vary widely, with some municipalities charging rent for the use of dental practices or not providing funds for the improvement or replacement of equipment (11).

Municipalities can only contract with dentists if they have a practice permit, which can be obtained in several ways (e.g., by purchase, exchange, or filling a newly established or previously vacant practice) (16). In addition, dentists can start providing services to the population only if they have an operating license and a contract to finance dental services. The National Health Insurance Fund (NHIF) can conclude the financing contract with the municipal government, the health care provider or the dentist (11).

Act LXXXIII of 1997 (17) regulates the scope of primary care dental services available under compulsory health insurance. Annual dental examinations, dental plaque removal, various preservation treatments (fillings, root canal treatment), dental surgical treatments and treatment of periodontal disease are available free of charge and without age restrictions (4, 13, 18). However, orthodontics, dental implant treatments, and fixed prostheses are available for a fee. People under 18 years of age, pregnant women and people over 62 are entitled to all dental services free of charge, but they have to pay for the technical costs (4, 13). Due to the limited range of services available free of charge under social insurance, the role of private care in dentistry has been growing for decades (18, 19).

### 1.4 Financing dental care in Hungary

From the early 1990s until 2019, the compensation of publicly financed primary care dental practices was based on two main pillars (20, 21). One was the fixed monthly fee based on the number of inhabitants in the area to be served and the age composition of the dental practice, which was calculated using a base financing method (22). The other main element was the performance-based payment, which was based on the number of interventions reported by the dental practice and the value of each intervention as determined by the NHIF (13). The minimum time required for different types of care was regulated for performance-based payments. Other minor funding parts included utility allowances, wage subsidies, and supplementary remuneration for practices in more disadvantaged municipalities.

During the COVID-19 pandemic, between 2020 and 2022, the payment of publicly funded primary dental care providers (alongside other primary care providers, specialized clinics and hospitals) was based on an average funding formula, taking into account the fixed and average performance fees of previous years (23). This financing method made it possible to plan and predict providers’ income during the different patient flows during the pandemic.

From 2023, primary dental care is financed using a new methodology. The fixed monthly fee based on base funding has been abolished, and the value of interventions eligible for performance funding has been increased (23). The new system, therefore, places more emphasis on the performance reported by dental practices (24). The minimum timeframes for interventions have been increased by 10% on average. In addition, an indicator system has been introduced whereby the proportion of people attending dental check-ups and the frequency of tooth extractions or fillings per practice within a certain period is assessed (23). Dental practices that perform above the average for a given indicator will receive additional remuneration from NHIF. The fees for services not covered by social insurance are reimbursed out of the patients’ own pockets or by the patient’s private health insurance (25).

### 1.5 Training and competencies of dentists in Hungary

University-level education in dentistry has been a tradition in Hungary for more than 150 years. The first faculty of dentistry was founded in 1955 (26). Currently, there are four medical universities in the country with a faculty of dentistry. University dental education lasts for 10 semesters, and graduates receive an MSc doctorate in dentistry (Doctor of Medicine in Dentistry), entitling them to work as independent patient care providers. Postgraduate specialized dental qualification programs of 36 months in dento-alveolar surgery, orthodontics, pediatric dentistry, periodontology, endodontics and prosthodontics are available after graduation (27). An appended further specialized qualification requiring an additional 24 months of training is available in oral implantology. A so-called vocational specialization license (license exam) is also available in the field of dento-maxillo-facial radiology, requiring an additional 18-months of training with a postgraduate specialist dental qualification or 12 months of training with a radiologist postgraduate specialist qualification (28) (Supplementary Table 1).

In Hungary, healthcare activities, including dental care, can be carried out independently or under supervision. Only those with the appropriate qualifications and registered in the operational register of healthcare workers (i.e., have a valid operational registry entry) may carry out healthcare activities independently (29). The NDGH keeps the operational register.

### 1.6 Collaboration between service providers

Primary care dental clusters were established on 14 June, 2021, based on Government Decree 53/2021 (9.II.) on primary care clusters (30). The main objective of the policy measure was to enable primary care dental practices to establish active professional cooperation within the dental clusters, to improve the population’s access to preventive dental services and to improve the quality of services by adapting methodological protocols developed by the NDGH. Practices participating in the dental clusters will receive additional funding and free professional training and conferences. The NDGH publishes a call to set up dental clusters once a year. According to the Government Decree 53/2021 (9.II.), dental clusters can be formed within a defined geographical unit (district), in the vicinity of each other, with at least 5 providers and without an upper limit. A primary care dental practice typically involves a dentist and two auxiliaries, mainly dental assistants. A dental cluster involves dentists and dental assistants working in primary dental practices.

Dentists can choose between three forms of collaboration:

1. The united district dental cluster is a close professional and economic cooperation between several primary care dental practices, whereby the participating dental service providers give up their economic independence and merge into a jointly established healthcare provider.
2. The integrated district dental cluster is a close professional and economic cooperation between several primary care dental practices. The participating dental service providers partly retain their economic autonomy and form a jointly established health care provider to perform their tasks co-ordinately.
3. The consortium of district dental cluster is a professional and economic cooperation between several primary care dental practices. The participating dental service providers, while fully retaining their economic autonomy, create a consortium cooperation agreement with each other in order to coordinate their tasks and appoint a consortium leader to represent the cooperation.

This study aims to describe the advancement of primary care dentistry in Hungary and the progress of the dental cluster model since 2021 legislation.

## 2 Methods

Our mixed-methods research used two different methodologies. First, a scoping review was conducted on the health policy developments of Hungary’s publicly funded primary dental care system. Second, secondary data analysis was carried out on developing primary care dentistry and dental cluster practices in Hungary.

### 2.1 The method of the scoping review

A scoping review was carried out in December 2023 to collect information on the health policy developments of Hungary’s publicly funded primary dental care system. The scoping review was performed according to the PRISMA guidelines, specifying the purpose of the study, the search strategy and keywords, the inclusion and exclusion criteria, the data selection and analysis method, and the results synthesis method.

**Figure 1:**
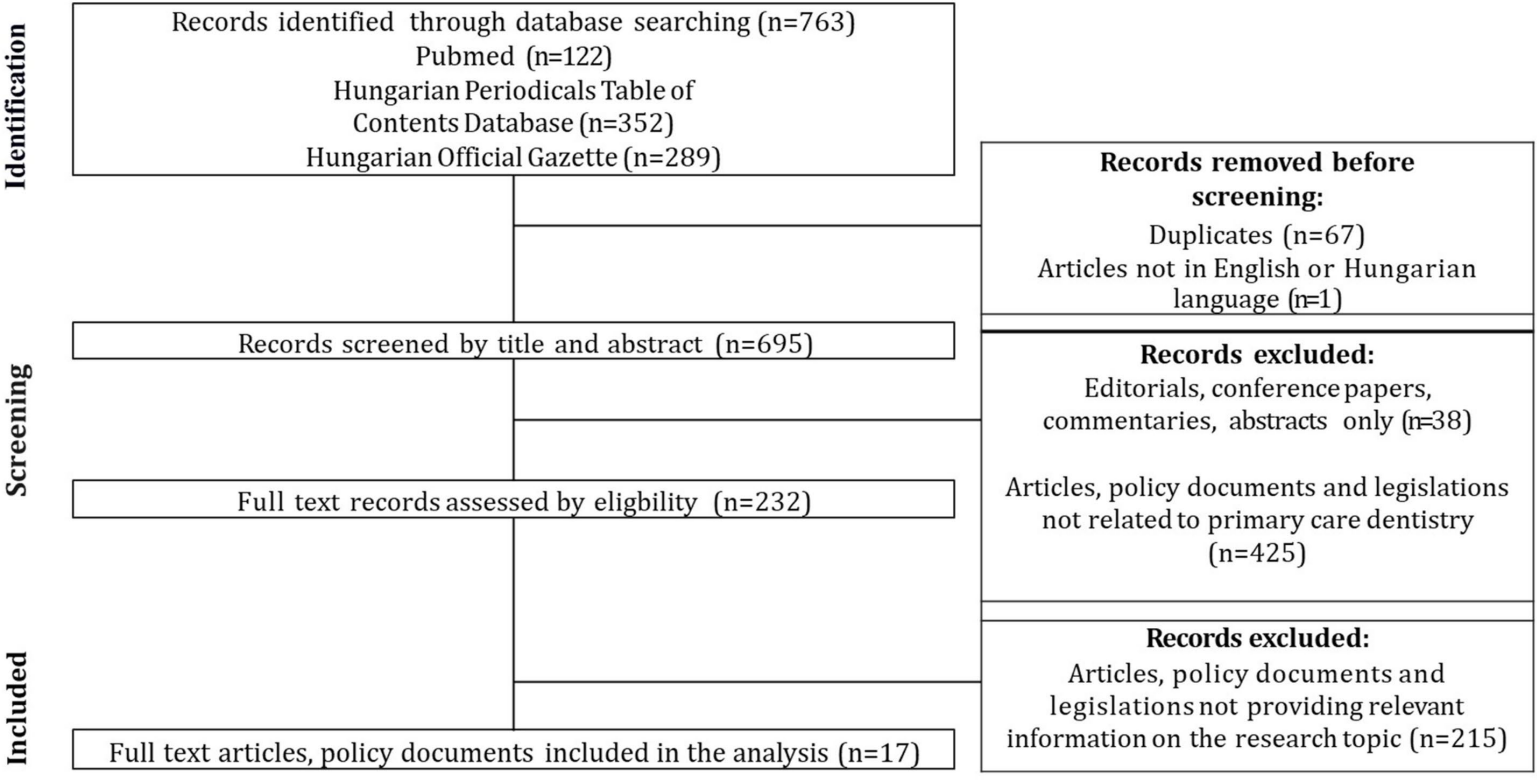
Flowchart of the results of the scoping review, according to the PRISMA guideline. Sources: Authors, based on the PRISMA guideline.

The literature search was performed using the PubMed search engine, the Hungarian Periodicals Table of Contents Database, and the Hungarian Official Gazette, utilizing the following keywords in Hungarian and English: dentist, dental care, primary care, practice, dental cluster, Hungary (Figure 1).

The selection criteria were that the full-text publication, strategy or legislation should contain relevant information on the health policy developments of Hungary’s publicly funded primary dental care system and be fully accessible in Hungarian or English. Exclusion criteria were publications published before 1990, commentaries, conference papers, editorials, abstract-only publications, or publications/legislations that did not contain relevant information on the health policy aspects of Hungary’s primary dental care system. Finally, the literature search resulted in 763 documents; after screening the titles, abstracts and full-text documents, 17 publications were included in the analysis. Documents were organized according to a coding frame with label definitions using Atlas.ti software. Two research team members coded in parallel, and the whole team interpreted the results.

### 2.2 The method of secondary data analysis

In January 2024, we requested data from the National Directorate General for Hospitals (NDGH) about Hungary’s primary care dentistry and dental clusters. The NDGH maintains and manages the 102 state-owned hospitals in Hungary and records healthcare human resources, their qualifications, and the actors involved in specialized and primary care. The request included the research design, the scope, time period and geographical coverage of the data requested, as well as the methodology of data analysis. Data and information on primary care dentistry were requested from NDGH on the following topics:

- for the period 1997–2022, the number of Hungarian and foreign nationals who graduated as a dentist in a given year, broken down by year;
- for the period 2010–2022, the number of dentists who are active and registered in the professional register, broken down by year and by qualification;
- for the period 2018 to 2024, the number of vacant and occupied primary care dental practices, broken down by year.

The data have been assembled in Excel spreadsheets. Data for primary care dentistry in Hungary from 1997 to 2023 were evaluated using descriptive statistical analysis. We described the number and proportion of Hungarian and foreign dentist graduates, active dentists in the professional register, by year and by qualification, and vacant and active primary care dentist practices.

Data and information on the operation of dental practice clusters have been requested from the NDGH on the following topics for individual practices and dental clusters:

- for the period 2021 to 2023, the number of dental clusters, broken down by year and geographical area, with the number of participating practices and with the unique funding identifier of each dental practice,
- for the period 2021 to 2023, the number of preventive services provided by the dental clusters, by geographical area and by year,
- for 2023, the number of dentists and health professionals working in dental clusters, by qualification types and by geographical area.

The NDGH database on the operation of dental practices was linked with the National Health Insurance Fund’s (NHIF) publicly available primary care dentist practice database as of December 2023, which included address information (such as municipality, district and county), practice status (active, vacant) and practice type information (adult, mixed) for individual dental practices. The individual practice data in the databases were linked using the unique NHIF funding identifier, and the data were cleaned using Stata Statistical Software 17.0 BE-Basic Edition. The generated database was further augmented with the Hungarian Central Statistical Office’s (9) publicly available municipal database for the year 2022, which included the type of municipalities, population data for municipalities and districts, a complex indicator of the level of development of districts, and counties. The databases were linked by the names of the municipalities.

The purpose of the regression analysis was to explore factors that could be associated with joining a dental cluster. Thus our dependent variable in this analysis was membership in the dental cluster (binary variable: joined/not joined). The independent variables included data about dental practices, the settlement where dental practices operate, and the district where the clusters operate. Practice-related information included practice type (adult/child/mixed), legal status of the settlement where the practice operates (village, small town, town, county town, county capital, capital districts), and population size of the settlement the practice is operating. The district is a geographic-administrative territory where primary care dental clusters may form and operate. District-level information included the level of social-economic development according to the official categorization of spatial development and planning (severely deprived, deprived, less developed, developed) as set out in Government Decree 290/2014 (XI. 26.) on the classification of beneficiary districts (31). District-level information also included the number of dentist practitioners per 10,000 inhabitants in the district.

For the descriptive results, we generated frequencies, means and 95% confidence intervals to describe the characteristics of practices who decided to join a dental cluster and those who did not. We ran bivariate and multivariate logistic regression models to examine the relationship between dentist and practice-related characteristics and the outcome. Bivariate analyses were performed to illustrate unadjusted differences between dentists who have become dental cluster members vs. non-members. We explored multivariate logistic regression models to understand the multiple factors that affected dentists’ likelihood of joining a primary care dental cluster. The final model was built by testing the inclusion of practice-related variables (sex, type, type of practice, type of settlement, number of settlement dentists), and then district-related variables (district development, district population). All study analyses were conducted using STATA v.17.0 BE-Based Edition and statistical significance was assessed at a *p*-value <0.05.

## 3 Results

### 3.1 The results of the scoping review

#### 3.1.1 Main health policy developments influencing primary care dentistry in Hungary between 1990 and 2023

From the early 1990s to 2024, several policy documents and legislation have been published, some of which have changed how primary dental care operates. The first strategy document, “Government Decision 1030/1994 (IV. 29.) on the principles of long-term health promotion policy,” was published at the end of the government term following the regime change in 1990 (32). The document set out general public health priorities and objectives and included a 20-point task list for 2000, but none addressed primary dental care. The implementation of some strategy elements was also delayed, mainly due to a lack of resources caused by the economic difficulties of the 1990s and the lack of operational plans (33).

The Public Health Program for a Healthy Nation 2001–2010 and the Decade of Health Public Health Program 2003–2013 already included several elements on primary dental care. Due to the poor oral health status of the Hungarian population, the development of preventive care and programs within primary dental care and the national expansion of the school dental network have been identified as a task. In addition, the ageing population of general practitioners and dentists has made it necessary to strengthen training systems to ensure an adequate supply of qualified and skilled human resources. GPs and dentists working in primary care were identified as partners in implementing action plans and tasks for public health programs. Many elements of the strategies and measures set out in these policy documents have also remained unimplemented, partly due to a lack of resources, a lack of the requirements for implementation, and insufficient cooperation and coordination between sectors and professions.

Between 1997 and 2000, several laws were passed that have fundamentally shaped how primary dental care has functioned up to the present day. In 1997, a law on the scope of primary care dental services available under compulsory health insurance came into force, and in 1999, a law described the rules for financing these services (16, 17, 21, 34). In 2000, laws and regulations were enacted regulating the legal status of general practitioners, general pediatricians and dentists to practice and the details of tasks and cooperation between local authorities and dentists (16, 34, 35).

The Healthy Hungary (2014–2020) Sectoral Strategy for Health published in 2014 recognized the ageing workforce of general practitioners and dentists in primary care and the resulting increase in the number and proportion of vacant GP and dental practices as a priority issue (36). The document’s situational assessment also identified considerable geographical inequalities, with some deprived districts having a vacancy rate of up to 30–40%, compared with economically developed districts where the rate is between 0 and 3%. The migration of doctors in the younger age group (30–40 years) to foreign countries was also identified as a problem, and scholarship schemes for medical residents were identified as an appropriate intervention to reduce this trend. The strategy also identified an essential role for primary care clusters, which can help reduce the workload of hospital care, mainly through collaboration with primary care providers and public health providers. The strategy also aimed to provide financial support for developing equipment and infrastructure in general medical and dental practices.

Between 2014 and 2017, the various primary care cluster models and methods were tested in pilot projects supported by the European Union and the Swiss Fund. The 2015 Act CXXIII of 2015 on primary health care (12) was created mainly due to these programs. According to the Act, primary care clusters can be established by general practitioners, general pediatricians, and dentists to provide primary care services to the population more efficiently and perform specific specialized care tasks. The detailed rules for establishing primary care clusters were laid down by law in a future decree, which was introduced in 2021.

The Healthy Hungary (2021–2027) Sectoral Health Strategy (37), published in 2021, focused on supporting the establishment of primary care clusters. The document reviewed the results of national programs modeling different ways of operating GP clusters. The strategy suggested that the initial successes of the model programs should be used to expand primary care clusters nationwide. The document summarized the legislation that defined the legal and organizational framework, the tasks of community practices, the primary care actors involved in the cooperation, the methods of financing and the scope of preventive services in community practices. In the strategy section assessing the sector’s human resources situation, it is noted that the number of dental assistants has decreased by 5% compared to 2010. In addition, an ageing dental assistant workforce was identified as a problem. The strategy identifies the need to improve the training system, salaries and working conditions to ensure sufficient human resources and promote dentistry among young people. The strategy identifies the application for resettlement of dentists to fill vacant dental practices as a good practice.

#### 3.1.2 The current operational model of the primary care dental clusters

**Figure 2:**
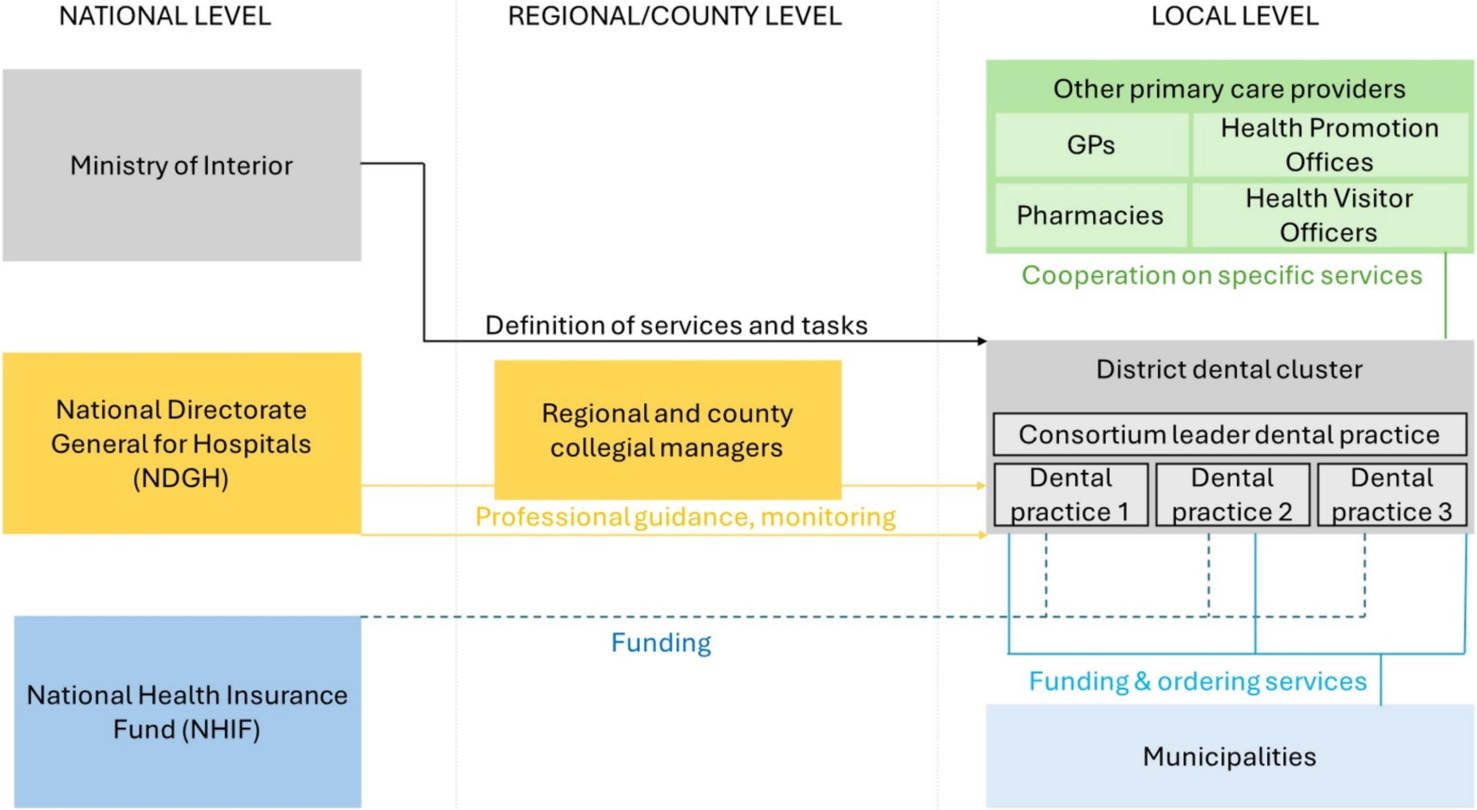
Simplified operational model of the primary care dental clusters in Hungary. Sources: Authors.

The NDGH carries out the professional management of the district dental clusters established in June 2021 under the coordination of the previously appointed county and regional collegiate managers. The dental cluster consortium leaders prepare a quarterly evaluation of the professional work carried out by their respective practices, which is shared with the collegiate leaders and the NDGH (Figure 2).

District dental clusters need to develop a service plan in which certain hours of preventive services should be provided based on the population of their service area. If the population is below 3,600, the preventive services should be at least 4 h per week. If the population exceeds 3,600, at least 5 h of weekly preventive services is necessary. The dental practices participating in the cluster must coordinate their schedule. If there is a permanent vacant dental practice in the district, the local municipality may request the dental cluster to cover the care of the residents in that area.

District dental clusters are recommended for developing partnerships with local health care providers, such as outpatient clinics, other primary health care providers, social care providers, municipalities, NGOs and professional organizations. Cooperation with public health service providers in the district, such as the Health Promotion Office, is also recommended.

The dental clusters also provide the NDGH with a range of data and information on their activities. They are required to produce a competency map of the education, training and practice time of the health professionals working in their organization. The clusters must also submit a competence development and equipment development plan, including the skills, licenses and qualifications the health professionals wish to acquire and the equipment and developments they wish to gain.

### 3.2 Results of the secondary data analysis

#### 3.2.1 Distribution of filled and vacant primary care practices in Hungary

Permanent vacant primary care practices are defined in Government Decree 313/2011 (XII. 23.) on the implementation of Act II of 2000 on the independent practice of medicine as a permanently vacant general practitioner district: a general practitioner district with a territorial coverage obligation, in which the obligation to provide care can be fulfilled only by substitution for a period of more than 6 months (34). The number of publicly funded primary care dental practices between 2018 and 2024 stagnated between 2,603 and 2,626 (Table 1). The proportion of vacant dental practices ranged between 9.2 and 11.2% over the period. There is an upward trend in the number and proportion of vacant adult dental practices (from 4.4 to 6.1%), and the proportion of vacant pediatric dental practices is also substantial, ranging from 13.5 to 17.8% between 2018 and 2024.

**Table 1:** Number and proportion of primary care dental practices and vacant practices 2018–2024 by practice type.

	2018	2019	2020	2021	2022	2023	2024
Number of primary care dental practices	2,619	2,620	2,606	2,613	2,626	2,611	2,603
Number and proportion (%) of primary care practices by practice type							
Adult	437 (95.6%)	442 (95.9%)	434 (95%)	432 (94.3%)	434 (95%)	436 (95%)	429 (93.9%)
Pediatric	154 (86%)	150 (83.3%)	148 (82.2)	151 (83.3%)	167 (86.5%)	166 (86.5%)	169 (86.2%)
Mixed	1,754 (88.5%)	1,757 (88.8%)	1,731 (87.9%)	1,740 (88.2%)	1,773 (89.7%)	1,768 (90.2%)	1,739 (89.2%)
All	2,345 (89.5%)	2,349 (89.7%)	2,313 (88.8%)	2,323 (88.9%)	2,374 (90.4%)	2,370 (90.8%)	2,337 (89.8%)
Number and proportion (%) of vacant primary care practices by practice type							
Adult	20 (4.4%)	19 (4.1%)	23 (5%)	26 (5.7%)	23 (5%)	23 (5%)	28 (6.1%)
Pediatric	25 (14%)	30 (16.7%)	32 (17.8%)	31 (17%)	26 (13.5%)	26 (13.5%)	27 (13.8%)
Mixed	229 (11.5%)	222 (11.2%)	238 (12.1%)	233 (11.8%)	203 (10.3%)	192 (9.8%)	211 (10.8%)
All	274 (10.5%)	271 (10.3%)	293 (11.2%)	290 (11.1%)	252 (9.6%)	241 (9.2%)	266 (10.2%)

Sources: NDGH and the authors.

**Figure 3:**
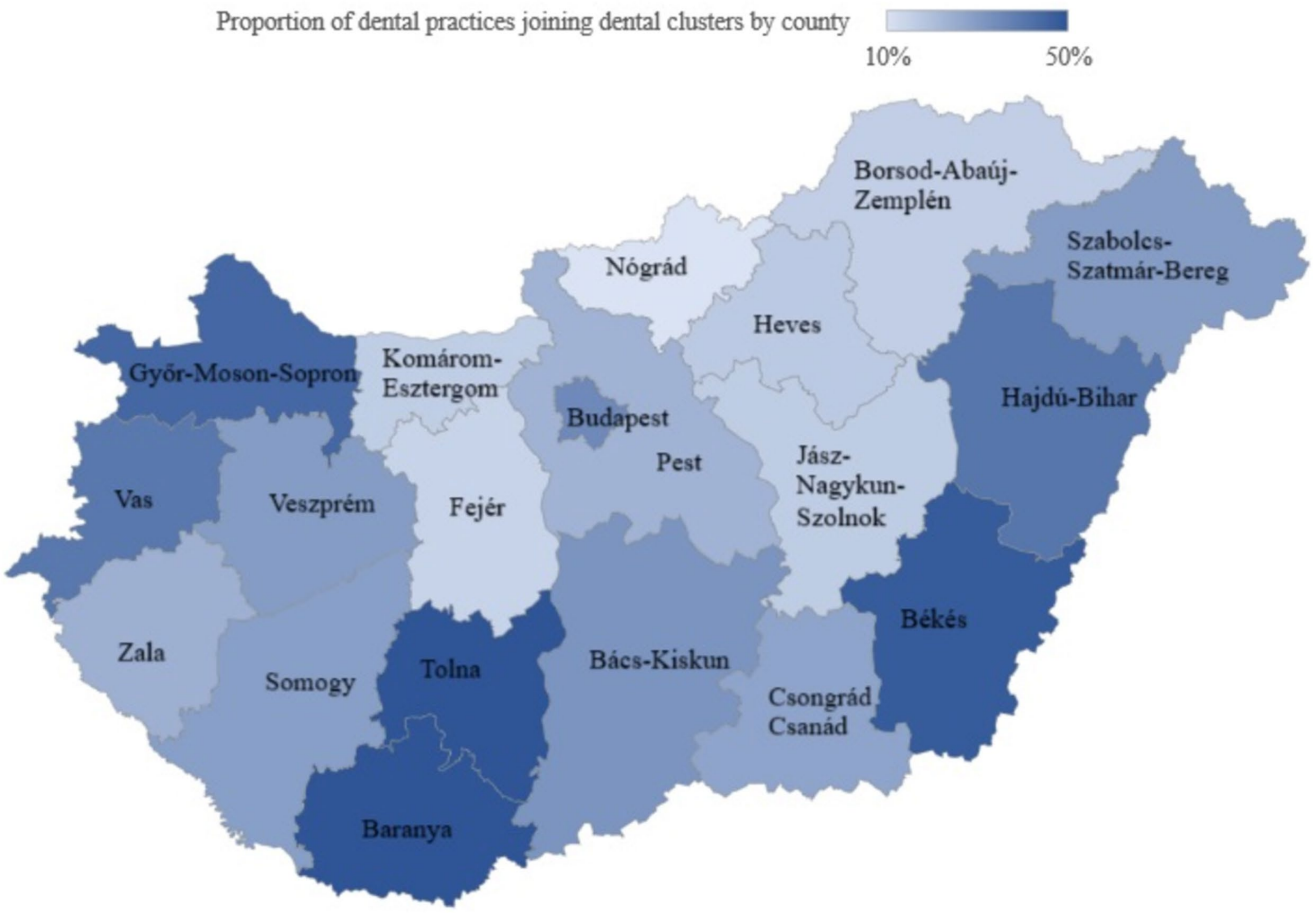
The proportion of dental practices joining district dental clusters by county in 2023. Sources: Authors.

By December 2023, 30% of dental practices nationwide were joined to district dental clusters, with 12 of the 19 counties below the national average (Supplementary Table 2). The lowest proportion of dental practices in dental clusters were in the counties of Nógrád (10%), Fejér (15%), and Borsod-Abaúj Zemplén (16%), and the highest in the counties of Békés (48%), Tolna (50%), and Baranya (50%; Figure 3). In Budapest, slightly above the national average, 35% of dental practices joined dental clusters.

#### 3.2.2 Trends in the training of dentists and the HR situation of dental clusters in Hungary

Between 1997 and 2022, the number of Hungarian and foreign nationals who obtained a dental degree in Hungary in a given year increased (Table 2). There were 67% more graduates in 2022 than in 1997 for Hungarian nationals, while the increase was nearly 28 times for foreign nationals. The increase in the number of foreign nationals is because Hungarian universities have started dentistry training in English and German in addition to Hungarian. In addition, it can be seen that some of the Hungarian and foreign dentists who graduated between 1997 and 2022 did not remain in the Hungarian healthcare system, as only 56–84% of the graduates in those years had a valid license in 2023, so some of the dentists are likely to have taken up employment in other EU or third countries in the years following graduation.

**Table 2:** The number of Hungarian and foreign nationals who have obtained a dental degree in Hungary between 1997 and 2022, and the number and proportion of Hungarian and foreign dentists with a valid professional registration on 31 December 2023, by year of graduation.

Year	Number of Hungarian nationals who have obtained a dental degree	Number and proportion (%) of Hungarian dentists with valid professional registration on 31 December 2023	Number of foreign nationals who have obtained a dental degree	Number and proportion (%) of foreign dentists with valid professional registration on 31 December 2023	Total number of dental graduates	Number and proportion (%) of all graduates with valid professional registration on 31 December 2023
1997	142	111 (78%)	5	2 (40%)	147	113 (77%)
1998	128	114 (89%)	8	0 (0%)	136	114 (84%)
1999	125	99 (79%)	8	0 (0%)	133	99 (74%)
2000	132	111 (84%)	43	1 (2%)	175	112 (64%)
2001	157	133 (85%)	34	0 (0%)	191	133 (70%)
2002	132	107 (81%)	26	1 (4%)	158	108 (68%)
2003	124	101 (81%)	35	0 (0%)	159	101 (64%)
2004	132	108 (82%)	22	0 (0%)	154	108 (70%)
2005	118	89 (75%)	15	0 (0%)	133	89 (67%)
2006	120	95 (79%)	35	0 (0%)	155	95 (61%)
2007	137	112 (82%)	36	0 (0%)	173	112 (65%)
2008	124	107 (86%)	40	1 (3%)	164	108 (66%)
2009	130	114 (88%)	62	3 (5%)	192	117 (61%)
2010	151	127 (84%)	82	4 (5%)	233	131 (56%)
2011	171	137 (80%)	82	5 (6%)	253	142 (56%)
2012	215	174 (81%)	96	7 (7%)	311	181 (58%)
2013	187	167 (89%)	115	8 (7%)	302	175 (58%)
2014	197	180 (91%)	134	15 (11%)	331	195 (59%)
2015	181	164 (91%)	127	20 (16%)	308	184 (60%)
2016	210	181 (86%)	139	24 (17%)	349	205 (59%)
2017	168	123 (73%)	141	23 (16%)	309	146 (47%)
2018	240	235 (98%)	150	88 (59%)	390	323 (83%)
2019	198	195 (98%)	123	63 (51%)	321	258 (80%)
2020	216	213 (99%)	115	59 (51%)	331	272 (82%)
2021	219	213 (97%)	105	55 (52%)	324	268 (83%)
2022	238	221 (93%)	138	49 (36%)	376	270 (72%)

Sources: NDGH and the authors.

Between 2010 and 2022, the number of dentists with various specialized dental qualifications in the NDGH’s registration database steadily increased (Table 3). However, some of the dentists were not working in the publicly funded health system or were not necessarily working in Hungary.

**Table 3:** Total number of dentists with a valid registration in the operational register of health professionals as of 31 December of the year under review and breakdown by the specialized dental qualifications for which they have a valid registration in the operational register.

Year	Total number of dentists	Dento-alveolar surgery	Endo-dontics	Prosthodontics	Orthodontics	Pediatric dentistry	Conservative dentistry and prosthodontics	Oral implantology	Periodontology	Dentists with no specialization
2010	5,137	219			304	209	4,289		42	74
2011	5,383	267			345	219	4,338		50	164
2012	5,770	301			381	226	4,516		62	284
2013	6,075	344			426	233	4,627		72	373
2014	6,398	384			483	240	4,765		86	440
2015	6,080	412			493	236	4,290		98	551
2016	6,342	456			529	253	4,398		111	595
2017	6,577	496			554	255	4,436		122	714
2018	6,868	527			583	258	4,525		130	845
2019	7,118	561	12	45	602	269	4,573	46	144	866
2020	6,581	557	31	67	559	223	3,908	54	151	1,031
2021	6,900	601	45	103	601	235	3,989	58	156	1,112
2022	7,198	648	56	149	642	245	4,020	61	167	1,210

Sources: NDGH and the authors.

The HR circumstances of the dental clusters displayed a mixed picture. By 31 December 2023, 74 dental clusters had been established in Hungary. 638 dentists and 653 dental assistants were employed in the dental cluster. The average number of dentists and dental assistants per dental cluster was 9. The 638 dentists had 776 specializations. The highest proportions were qualified as doctors of dental and oral diseases (66.9%), dentists (19.9%) and conservative dentistry and prosthodontics (18.5%; Table 4). 73% of dentists had one specialization, 22% had two, 4% had three and 1% had four (Supplementary Table 3).

**Table 4:** Specializations of dentists working in dental clusters in 2023*.

Specializations	Number and proportion of dentists specialized as a percentage of all dentists
General medicine for oral diseases	20 (3.1%)
Pathology	6 (0.9%)
Surgery	3 (0.5%)
Psychotherapy	1 (0.2%)
Doctor of dental and oral diseases	427 (66.9%)
Pediatric dentistry	13 (2.0%)
Orthodontics	23 (3.6%)
Oral surgery	5 (0.8%)
Social medicine	2 (0.3%)
General medicine	3 (0.5%)
Dentist	127 (19.9%)
Dento-alveolar surgery	15 (2.4%)
Periodontology	5 (0.8%)
Orthodontics	2 (0.3%)
Conservative dentistry and prosthodontics	118 (18.5%)
Pediatric dentistry and orthodontics	3 (0.5%)
Traditional Chinese medicine	3 (0.5%)

*If one dentist has more than one specialization, he/she is listed more than once in the table. Sources: NDGH and the authors.

The 653 dental assistants had a total of 932 qualifications. The highest proportions were dental assistants (90.0%), clinical dental hygienists (24.2%) and general nurses and assistants (8.0%) (Table 5). 63.2% of dental assistants had one specialization, 28.5% had two, 6.6% had three and 1.4% had four (Supplementary Table 4).

**Table 5:** Specializations of dental assistants working in dental clusters in 2023*.

Qualification	Number and proportion of dental assistants qualified as a percentage of all dental assistants
General nurse	33 (5.1%)
General nurse and assistant	52 (8.0%)
General assistant	14 (2.1%)
Infant and child nurse	12 (1.8%)
Graduate nurse	3 (0.5%)
Laboratory assistant	4 (0.6%)
Radiographer	1 (0.2%)
Dental assistant	588 (90.0%)
Dental assistant, oral hygienist	38 (5.8%)
Dental technician	9 (1.4%)
Clinical dental hygienist	158 (24.2%)
Pharmacy assistant	6 (0.9%)
Physiotherapy assistant	5 (0.8%)
Massage therapist	2 (0.3%)
Midwife	3 (0.5%)
Addictologist	1 (0.2)
Surgical technician	1 (0.2%)
Public health official	2 (0.3%)

*If one dental assistant has more than one specialization, he/she is listed more than once in the table. Sources: NDGH and the authors.

#### 3.2.3 Description of the preventive services provided by dental clusters

In 2022, the dental clusters reported to the NDGH the provision of 11 different preventive services, with a total of 83,423 preventive services provided in the year (Table 6). Of these, the highest proportions reported were caries intensity measurements (34.5%), screening for oral cancer (23.2%), and screening for TMJ problems (15.2%).

**Table 6:** Number of preventive services accounted by dental clusters in 2022.

Service	Number and proportion of preventive services reported by dental clusters (%)
Microbiological testing (e.g., COVID, RSV, Streptococcus A)	5 (0.01%)
Screening people at risk of diabetes mellitus with Findrisk questionnaire	83 (0.10%)
Screening people at risk of diabetes mellitus with Findrisk questionnaire and rapid test	5 (0.01%)
Health literacy survey among the adult population/Health literacy parental support (pediatrician practice)	936 (1.12%)
Smoking dependence screening, minimal intervention	392 (0.47%)
Screening for alcohol dependence, minimal intervention	205 (0.25%)
Screening for oral cancer	19,534 (23.42%)
Screening for periodontal diseases, using periodontal screenings and indices	13,036 (15.63%)
Measuring caries intensity (caries prevalence) in the population	28,819 (34.55%)
Dental anamnesis at the age of 0–18 years	7,682 (9.21%)
Detecting jaw joint problems	12,726 (15.26%)
All	83,423 (100%)

Sources: NDGH and the authors.

#### 3.2.4 Results of the regression analysis to explore factors that could be associated with joining a dental cluster

The characteristics of dental practices by dental cluster membership is shown in Table 7. Altogether, there were 2,491 dental practice units, out of which 227 were vacant practices, i.e., no practizing dentists for more than 6 months (34). The total sample in the analysis was 2,264.

**Table 7:** Characteristics of dental practices by dental group practice membership.

Characteristics	N /proportion % (95% CI) or mean (SD; min-max)		Total
Variables	Joined a dental group practice	Not joined a dental group practice	N / % (CI) or mean
Dental practice	750 /33% (CI: 0.31–0.35)	1,514 /66.9% (CI: 0.65–0.69)	2,264
Sex			
Male	280/37.3% (CI: 0.34–0.41)	571 /37.7% (CI: 0.35–0.41)	851 /37.5% (CI: 35.6–39.6)
Female	470 /63% (CI: 0.59–0.66)	943 /62% (0.59–0.65)	1,413 /62.4% (CI: 60.39–64.3)
*Practice type			
Pediatric	26 /3.47% (CI: 0.02–0.05)	136 /8.98% (CI: 0.07–0.10)	162 /7% (CI: 6.1–8.3)
Mixed	541 /72% (CI: 0.69–0.75)	1,135 /75% (CI: 0.73–0.77)	1,676 /74% (CI: 0.72–0.76)
Adult	183 /24.4% (CI: 0.21–0.28)	243 /16% (CI: 0.14–0.18)	426 /18% (CI: 0.17–0.20)
*Settlement type			
Village	111 /14% (CI: 0.12–0.17)	302 /19.9% (CI: 0.18–0.22)	413 /18% (CI: 0.16–0.2)
Small town	37 /4.9% (CI: 0.04–0.07)	94 /6.2% (CI: 0.05–0.07)	131 /5.7% (CI: 0.48–0.62)
Town	228 /30.4% (CI:0.27–0.34)	556 /36.7% (CI: 0.34–0.39)	784 /34% (CI: 0.32–0.36)
County town	30 /4% (CI: 0.029–0.06)	45 /3% (CI: 0.02–0.04)	75 /3% (CI: 0.026–0.04)
County capital	183 /	259 /17% (CI: 0.15–0.19)	442 /19.5% (CI: 0.18–0.21)
Capital district	24.4% (CI: 0.21–0.27)	258 /17% (CI: 0.15–0.19)	419 /18.5% (CI: 0.17–0.20)
**Settlement population size	750 /Mean: 52528.7 (CI: 48652–56,405) (SD: 54080) (Min: 391 – Max: 201582)	1,514 /Mean: 43059.5 (SD: 52789.2) (CI: 40398–45,721) (Min: 322–201,582)	2,264 /Mean: 46196 (SE: 1122) (CI: 43995–48,397)
District development level			
Severely deprived	63 /8.4% (CI: 0.067–0.11)	141 /9.31% (CI: 0.08–0.11)	204 /9% (CI: 0.078–0.10)
Deprived	26 /3.47% (CI: 0.02–0.05)	73 /4.82% (CI: 0.004–0.06)	99 /4% (CI: 0.036–0.05)
Less developed	138 /18.4% (CI: 0.16–0.21)	313 /20.6% (CI: 0.18–0.23)	451 /19% (CI: 0.18–0. 22)
Developed	523 /69.7% (CI: 0.66–0.73)	987 /65.2% (CI: 0.68–0.67)	1,510 /66% (CI: 0.64–0.68)
**No. of dentist practitioners per 10.000 people at district level	750 /Mean: 2.49 (SD: 0.57) (Min: 1.2 – Max: 5.7)	1,514 /Mean: 2.39 (SD: 0.61) (Min: 0.3 – Max: 5.8)	2,264 /Mean: 2.42 (SE: 0.012) (CI: 2.39–2.44)

*p-value for Chi2 test significant if < 0.05. **p-value for Student test significant if < 0.05.

In the study sample, there were 884 settlements and 196 districts in the sample. There were 750 practices who joined a dental cluster. Compared to dental practices who did not join a cluster, dental cluster members had a higher proportion of adult practices (adult 24.4% vs. 16%) and fewer pediatric practices (3.47% vs. 8.98%); they tended to come from cities with more population and with higher legal status (county town, county capital, capital districts); from more populated districts and districts with higher density of dentists. No significant differences were shown between dental cluster members and non-members for the variables of sex of dentists and district development level in the descriptive analysis.

The results of the bivariate and multivariate regression analyses are shown in Table 8.

**Table 8:** Unadjusted and adjusted ORs in the final model.

Independent variable	Unadjusted OR / (95% CI)	*p*-value	Adjusted OR / (95% CI) (adjusted for all other variables)	*p*-value
Sex (reference male)				
Female	1.01 (CI: 0.85–1.215)	0.88	1.02 (CI: 0.84–1.23)	0.819
Dental practice type (Reference: Pediatric)				
Mixed	2.49 (CI: 1.62–3.84)	0.000	4,13 (CI: 2.49–6.86)	0.000
Adult	3.94 (CI: 2.48–6.28)	0.000	3.88 (CI: 2.43–6.19)	0.000
Settlement type (Reference: village)				
Small town	1.07 (CI: 0.69–1.66)	0.75	1.07 (CI: 0.69–1.67)	0.74
Town	1.11 (CI: 0.85–1.45)	0.42	1.19 (CI: 0.911–1.567)	0.198
County town	1.81 (CI: 1.08–3.02)	0.02	2.30 (CI: 1.34–3.96)	0.002
County capital	1.92 (CI: 1.44–2.56)	0.000	2.93 (CI: 1.80–4.76)	0.000
Capital district	1.69 (CI: 1.26–2.27)	0.000	2.877 (CI: 1.705–4.855)	0.000
Settlement population size (No. of people)	1.000003 (CI: 1.000002–1.000005)	0.000	0.99 (CI: 0.99998–0.999997)	0.114
District development level (Reference: severely deprived)				
Deprived	0.79 (CI: 0.46–1.36)	0.40	0.806 (CI: 0.47–1.38)	0.434
Less developed	0.98 (CI: 0.69–1.41)	0.94	0.94 (CI: 0.655–1.357)	0.753
Developed	1.18 (CI: 0.86–1.62)	0.28	0.83 (CI: 0.59–1.18)	0.322
Number of dentist practitioners /10.000 inhabitants at district level	1.30 (CI: 1.12–1.500)	0.000	1.18 (CI: 1.01–1.37)	0.028

Sources: Authors.

After controlling for all other variables, sex had no statistically significant independent effect on the likelihood of joining a dental cluster in our analysis. As for dental practice type, mixed and adult practices were around four times more likely to join a dental cluster than pediatric practices [OR: 4.13 (CI: 2.49–6.86) and OR: 3.88 (CI: 2.43–6.19), respectively]. Settlement type was also independently associated with joining a dental cluster. Practices operating in county towns, county capitals and the capital districts were statistically significantly more likely to form dental clusters than those who work in villages. Practices in small towns and towns did not statistically differ from practices in villages. Settlement population size did not show a statistically significant independent association with joining a dental cluster. In our analysis, the district development level did not show a significant association with the likelihood of joining a dental cluster. Dentist density at a district level had a positive association with joining a dental cluster [OR: 1.18 (95%CI: 1.01–1.37)], dental practices in more populated districts were more likely to join a dental cluster.

## 4 Discussion

### 4.1 Oral health of the Hungarian population in international comparison

Oral health is a central part of overall health. WHO points out that oral diseases share the same major risk factors as common NCDs and they can be prevented and treated yet are among the most widespread conditions in Europe. The age-standardized prevalence of oral diseases is around 60% in Hungary, which is lower than in several European countries (4). The prevalence of major oral diseases (caries of untreated deciduous and permanent teeth, tooth loss, severe periodontal disease and other oral disorders combined) is estimated to grow further in Europe and in Hungary with the population ageing. According to international reports, the oral health of the Hungarian population along specific indicators (lip and oral cancer incidence, lip and oral cancer mortality, carries of permanent teeth) is alarming (8). At the same time, the level of unmet dental needs in Hungary was below the EU average of 4.7% (6). While Hungarians with low income are more likely to report unmet medical needs, income inequalities in Hungary are smaller than the EU average in unmet self-reported dental needs (1).

### 4.2 Dental health workforce in Hungary in international comparison

The health workforce is an important pillar of primary health care. The WHO report “Health and Care Workforce in Europe: Time to Act” identified the main challenges in the European Region, including ageing of the medical workforce, uneven distribution of health workers, personnel shortages, retention and recruitment challenges in rural areas, insufficient supply of new graduates, and lack of data to inform health workforce planning (38). According to Hungarian and international comparative data, workforce shortage has been a problem in Hungary for many years, just like in other European countries. The number of doctors per 1,000 population increased marginally from 2012 to 2021 (from 3.0 per 1,000 population to 3.3 per 1,000), lower than the EU average of 4.1 per 1,000 (1). In 2021, there were 5.3 nurses per 1,000 population, well below the EU average of 8.5 per 1,000. OECD points out that in many countries, the main concern has been about growing shortages of general practitioners, particularly in rural and other under-served areas (39). In EU countries, the density of physicians is greater in urban areas than in rural areas. According to the OECD report, the rural and urban differences in the density of doctors were especially large in Hungary, the Slovak Republic, Lithuania and Latvia, according to OECD. The same report also points out that the share of general practitioners of all the practicing doctors was one of the lowest in Hungary (12%) compared to the EU24 average of 20%.

The ageing of doctors is also a growing problem due to retirement and the pipeline to replace the retiring workforce. Between 2010 and 2020, Hungary was among the EU countries where the number of dentists per capita increased steadily (1).However, Hungary had a lower than EU average number of practicing dentists in 2021 (71 per 100,000). Moreover, annual dentist consultation per capita in 2019 and 2020 was among the lowest in Hungary (in 2019, 0.7 per person) (39). In the period of 2011–2021, the number of dentist graduates per population somewhat increased in Hungary, while in 2021, the level of graduating dentists was around the EU average (40).

### 4.3 Comparison of the Hungarian financing practices of primary dental care with international practices

Several international studies have shown that, compared to Western and Central European countries, Hungary has very low reimbursement and per capita public expenditure on outpatient dental care in publicly funded primary dental care, which is due, among other things, to the underfunding of the care system (13, 39).

According to the OECD 2022 analysis, the source of financing for dental care in Hungary is predominantly from out-of-pocket and voluntary health insurance and only about 30% from public spending (39). These proportions align with most other OECD countries, which also have a high share of private spending (above 50%). In 2019, Hungary had one of the highest proportions of families among the EU24 Member States, with catastrophic health expenditure in the past year (12%), mainly affecting families in the lowest quintile (39). In 2021, 12% of total health expenditure in Hungary was spent on primary care services, slightly lower than the average for OECD countries (13%). However, Hungary spent 8% of total health expenditure on preventive services, compared to an average of 5% for OECD33 countries.

### 4.4 Comparison of the operation of the Hungarian dental clusters with international practices

Like Hungary, several other European countries have started developing closer cooperation and clusters between primary care providers, such as dentists, dental hygienists and general practitioners. During the COVID-19 pandemic in the United Kingdom, dental clusters were established to provide services to a population of a defined geographical area, with different primary and specialist care providers working together (41, 42). The UK dental clusters, like the Hungarian clusters, aimed to improve access to primary care dental services, improve preventive approaches and reduce geographical inequalities. Another priority was to develop the knowledge and skills of professionals working in dental practices through joint work and mentoring. In contrast to the Hungarian dental clusters, a further objective was to develop multidisciplinary collaborations and approaches to meet the needs of the population. Therefore, the UK dental clusters were formed by primary care dental practices working with specialist dental care and surgery, GP practices and pharmacists. In contrast, in Hungary, only dental practices have formed clusters (41, 42) have shown that dental clusters can support the development of access to services for the population and cooperation between different primary care actors. Clusters provide significant professional development opportunities for dentists through regular professional meetings, internal training and case discussions. The human resources of dental clusters, with their wide range of expertise, also provide the opportunity to provide services to patients with complex needs within the primary care setting, avoiding the need for higher progressive levels of care.

Research by Boer and colleagues in the Netherlands has shown that the development of collaborative and preventive services among dental practices has increased in recent decades (43, 44). As in Hungary, changes in the Netherlands have been driven by several factors, such as changes in population demographics, the development of the dental profession, and various policies and regulations. While the size of dental practices has steadily increased with the expansion of different professional units within the practice, the number of practices has decreased in the Netherlands (44). Boer et al.’s research found that the collaboration between dentists and dental hygienists was mainly influenced by management style and the goals of the collaboration. For both factors, highly diverse practices emerged in the Netherlands, with professional and patient-focused arguments emerging among collaboration goals, while leadership practices also proved to be heterogeneous. There are both explicitly hierarchical and less hierarchical forms of governance of collaborative leadership across practices. Larger practices and collaborations had a higher degree of standardization of processes.

In a qualitative study in Germany on the opportunities and challenges of collaboration between dentists and general practitioners, it was found that there are few examples of collaboration between general practitioners and dentists, despite the many benefits for definitive patient care and prevention (45). Although dentists and general practitioners reported a lack of information about the other profession’s field and practices that may affect patient care, the willingness to collaborate was higher among dentists than general practitioners. Similar incomplete cooperation between dental and general practitioner providers is typical in Hungary, so it would be useful to investigate the reasons for this and identify opportunities and solutions to encourage cooperation. The results of Australian research confirm that collaboration between dental practices and primary care services can significantly improve access to services for people living in rural areas (46). Collaboration can provide opportunities for broader access to preventive and health promotion services, integrated patient pathways and more flexible service practices (47, 48).

### 4.5 Health policy and practical implications of the results

We found that mixed and adult dental practices, those operating in county cities, county capitals and capital districts, and those operating in districts with more dentists were likelier to join dental clusters. These relationships may caution a potential source of inequality in the access to strengthened primary care services between the different types of settlements and dental practices that health policy decision-makers could consider when evaluating the implementation of dental clusters. Health policymakers should, therefore, evaluate what incentives could be used to support dental practices from villages and small towns to join the cluster model.

### 4.6 Limitations and future areas of research

Our research has the following limitations. First, data on dentist training were only available from the NDGH for a limited period (1997 to 2022). Second, data on the number of preventive services provided by dental clusters were only available for 2021 and 2022, so we only had information on the initial period of the formation of dental clusters. Furthermore, we could not compare the preventive services of the practices with the data from the period before the clusters were established. No data were available on the dental clusters’ asset and infrastructure conditions and development plans, so we could not provide an analysis in this regard. No data and information were available on the number of collaborations established by clusters, their partners, or the themes of the collaborations. We also did not know why some dentists joined clusters and others stayed away. We plan to gather more information on this topic and the experience of developing dental clusters through qualitative research.

Furthermore, our multivariate logistic regression analysis had some important limitations. We did not have an ideal set of data on factors that may be associated with joining a dental clusters (as suggested by scientific literature). For example, we did not have data on the age of dentists and/or the dentist’s years in practice. We did not have data on practice size or age and/or socio-economic composition of the population at the practice level. Our multivariate analysis did not consider the potential clustering of observations within geographic/administrative units meaningful for primary dental practice or dental cluster operations such as settlements, districts and counties due to insufficient sample size for multilevel modeling.

Future research areas can be identified on the impact of the COVID-19 pandemic on the functioning of primary dental care in Hungary, as several national and international studies have shown that the pandemic has negatively affected the functioning of health systems and the population’s access to services (19, 49, 50). It would also be practical to investigate the potential for the widespread use of digital health technologies in primary dental care in Hungary (51). Another worthwhile area of research is to explore the potential for closer collaboration between dentists and other primary care actors, such as GPs and Health Promotion Offices (52).

## 5 Conclusion

This study allowed us to explore the development of primary care dental clusters in strengthening primary health care. The available literature emphasized that developing group practices or clusters between primary care providers can result in improved satisfaction and quality of care, improved quality of life and income for physicians, and improved efficiency and better utilization of resources for health care systems (53, 54). This study explored the available literature on the health policy and legislative developments supporting and regulating the establishment of Hungary’s publicly funded primary dental care system. We identified that until the end of 2023, nearly one-third of the dental practices participated in a district dental cluster. We also identified regional differences in the percentage of dental practices who joined a district dental cluster. This study also explored the potential factors influencing the act of joining a dental cluster. Our results suggest that mixed and adult dental practices, dental practices operating in county cities, county capitals and capital districts, as well as dental practices operating in districts with more dentists were more likely to join a dental cluster. These relationships may caution a potential source of inequality in the access to strengthened primary care services between the different type of settlements and the different type of dental practices that decision makers could consider when evaluating the implementation of dental clusters. We did not identify any information about the goals or expectations of policymakers regarding the pace of implementation and the optimal rate of participating dental practices. We can identify some interesting areas for further investigation: the impact of group practices on physicians, addressing patient outcomes and perspectives and impact on the health system, and identifying factors that make a primary dental care cluster’s operation efficient. Acknowledging that this study was prepared at an early stage of implementation of dental clusters in Hungary, monitoring and evaluation of primary care dental clusters’ operation, performance and impact in the future will be important.

## Data Availability

The data analyzed in this study is subject to the following licenses/restrictions: the study and the data provision have been authorized by the Ministry of the Interior on the basis of the research application BMTSKF/1–61/2024, approved on 9 April 2024. All relevant data may be obtained from the corresponding author upon a reasonable request. Requests to access these datasets should be directed to Csilla Kaposvári, kaposvari.csilla@semmelweis.hu.
