# Supplementary material for: The advancement of primary care dentistry in Hungary: progress of the dental cluster model since 2021 legislation
Source: Front Public Health. 2025 Jan 22;13:1528433. doi: 10.3389/fpubh.2025.1528433 (PMC11794789; doi:10.3389/fpubh.2025.1528433)
Supplement: Supplementary file 1 [file Table_1.DOCX]

Appendix 1. The development of primary care dental cluster practice model - experiences from Hungary. Supplementary Tables

András Sztrilich, Csilla Kaposvári, Gergő Túri, Rita Teller, István Vingender

*** Correspondence:**Csilla Kaposvári
[kaposvari.csilla@semmelweis.hu](mailto:kaposvari.csilla@semmelweis.hu)

**Supplementary Table 1.** Training and qualifications of the paramedical members of the dental team

| **Vocational training for a job or activity which does not require tertiary qualifications** | | | | |
| --- | --- | --- | --- | --- |
| **Sector** | **Profession** | **Entry requirements** | **Length of training w. high-school diploma/w technical college** | **Competences** |
| Medical technology | Dental technician | Upper secondary school diploma | 3 years/- | The dental technician works in a dental laboratory with appropriate equipment. In partnership and cooperation with the dentist and dental technician colleagues.  The work is based on professional statics, material science, dental functional anatomy, gnathology, aesthetics, phonetics and hygiene. The dental technician may carry out the following dental prostheses and prosthodontic work: removable plastic base plate and combined metal plate prostheses, framework and veneering of fixed prostheses, aesthetic dental solutions, metal-free prostheses, combined work, implant prostheses, orthodontic appliances, restorative prosthetic appliances, repair of various dental solutions. |
| Health care | Dental assistant (health care assistant) | Upper secondary school diploma or technical college diploma | 2 years/5 years | A dental assistant assists with everyday dental treatments and procedures under the direction and supervision of the dentist. He/she participates in the preparation of general and special dental treatments, directly assists the dental team members in the patient's care during the procedures and assists the dentist. Operates dental unit equipment and various specialized dental machines and equipment according to manufacturer's specifications. Ensures the repair and replacement of dental instruments, materials and equipment. Provides health education and preventive dental education to patients. |
| **Specialized vocational qualifications appended to vocational training** | | | | |
| Sector | Vocational training | Entry requirements | Length of training | Competences |
| Medical technology | Dental designer | Upper secondary school diploma  + dental technician diploma | Minimum 160 hrs/module or apprenticeship | The dental designer works in a dental laboratory using digital equipment, machines and associated software. The work is carried out on the basis of professional statics, material science, dental functional anatomy, gnathology, aesthetics, phonetics and hygiene, using computer-assisted 3D design, computer-controlled milling and printing. |
| Medical technology | Orthodontics | Upper secondary school diploma  + dental technician diploma | Minimum 160 hrs/module or apprenticeship | Orthodontic technicians work in dental laboratories to make orthodontic appliances, orthodontic appliances, extraoral appliances, appliances for functional muscle therapy and for the correction of speech defects, protective braces for sportsmen and women, preventive orthodontic appliances for children and adults, and orthodontic appliances for the treatment of speech defects. Devices and prosthetic solutions are designed in collaboration with the orthodontic dentist and dental technicians. |
| **Vocational qualifications defined in the National Training Register valid before 1 January 2020 being in phasing-out status:** | | | | |
| Health care | Clinical dental hygienist | Upper secondary school diploma + dental assistance | 1 year or minimum 450 hrs without dental assistant training | The clinical dental hygienist is a member of the dental team alongside the (specialist) dentist, dental assistant and dental technician. He/she shall perform his/her activities under the indication and supervision of a dental hygienist and under his/her own authority and responsibility. He/she is responsible for the dental health education and guidance of patients. |

Sources: EMMI (Decree 22/2012 (IX. 14.) and the Authors

**Supplementary Table 2.** Number and proportion of dental practices joined to the dental clusters by county, 2023.

| County | Number of dental practices | Number of dental practices joined to dental clusters | Proportion of dental practices joined to dental clusters |
| --- | --- | --- | --- |
| Nógrád | 59 | 6 | 10% |
| Fejér | 96 | 14 | 15% |
| Borsod-Abaúj-Zemplén | 174 | 28 | 16% |
| Komárom-Esztergom | 67 | 11 | 16% |
| Jász-Nagykun-Szolnok | 90 | 15 | 17% |
| Heves | 92 | 16 | 17% |
| Pest | 289 | 68 | 24% |
| Zala | 78 | 19 | 24% |
| Csongrád-Csanád | 118 | 33 | 28% |
| Somogy | 65 | 19 | 29% |
| Veszprém | 77 | 23 | 30% |
| Szabolcs-Szatmár-Bereg | 123 | 37 | 30% |
| National | 2491 | 750 | 30% |
| Bács-Kiskun | 143 | 46 | 32% |
| Budapest | 460 | 161 | 35% |
| Vas | 62 | 25 | 40% |
| Hajdú-Bihar | 135 | 55 | 41% |
| Győr-Moson-Sopron | 109 | 49 | 45% |
| Békés | 102 | 49 | 48% |
| Baranya | 102 | 51 | 50% |
| Tolna | 50 | 25 | 50% |

Sources: NDGH and the authors

**Supplementary Table 3.** Number and proportion of dentists with 1 or more specializations working in dental clusters in 2023

| Number of specialization | Number and proportion (%) of dentists with specializations |
| --- | --- |
| 1 | 464 (73%) |
| 2 | 142 (22%) |
| 3 | 26 (4%) |
| 4 | 6 (1%) |

Sources: NDGH and the authors

**Supplementary Table 4.** Number and proportion of dental assistants with 1 or more specializations working in dental clusters in 2023

| Number of qualifications | Number and proportion (%) of dental assistants with qualifications |
| --- | --- |
| 1 | 413 (63,2%) |
| 2 | 186 (28,5%) |
| 3 | 43 (6,6%) |
| 4 | 9 (1,4%) |
| 5 | 2 (0,3%) |

Sources: NDGH and the authors
